# Nomophobia-Related Indicators in Court-Involved Adolescents With Child-to-Parent Violence: A Preliminary Item-Level Correlational Study

**DOI:** 10.62641/aep.v54i4.2316

**Published:** 2026-08-15

**Authors:** Elisa González-Pérez, José Luis Díaz Palencia, Isabel Martínez-Álvarez

**Affiliations:** ^1^Faculty of Psychology and Health Sciences, Universidad a Distancia de Madrid (UDIMA), 28400 Collado Villalba, Madrid, Spain; ^2^Faculty of Education, Universidad a Distancia de Madrid (UDIMA), 28400 Collado Villalba, Madrid, Spain

**Keywords:** nomophobia, child-to-parent violence, adolescents, mobile phone, addiction

## Abstract

**Background:**

Nomophobia, commonly understood as fear or distress associated with being without access to a mobile phone, has become a relevant topic in adolescent research. However, little is known about how nomophobia-related indicators are associated with one another in adolescents involved in juvenile justice interventions for child-to-parent violence (CPV).

**Methods:**

This exploratory cross-sectional study included 53 adolescents serving court-ordered measures for CPV in a specialized juvenile justice centre in Madrid, Spain. Six items from the nomophobia subscale of the Risk Scale for Adolescent Addiction to Social Networks and the Internet were analyzed. Item-level bivariate correlations were examined, and the findings were interpreted cautiously in light of the exploratory nature of the study.

**Results:**

The six items showed positive associations, with stronger correlations among indicators related to immediate replies, read-receipt confirmation, restlessness when interaction is absent, and anger when separated from the mobile phone. Items referring to perceived safety when sending messages or photographs showed weaker associations with the remaining items.

**Conclusions:**

The findings suggest a preliminary pattern of internal association among six nomophobia-related Adolescent Social Media and Internet Addiction Risk Scale Reliability and Validity (ERA-RSI) items in this specific sample of court-involved adolescents. These results should not be interpreted as evidence of the full structure of nomophobia or as causal evidence regarding family conflict or interpersonal control. Future studies should use larger multi-site samples, validated nomophobia-specific instruments, longitudinal designs, and analytic strategies appropriate for ordinal data.

## Introduction

The expansion of mobile digital environments has transformed adolescent communication by intensifying immediacy, permanent availability, and dependence on mobile connectivity. Within this context, nomophobia has emerged as a relevant construct, generally understood as fear, discomfort, or anxiety associated with being unable to access or use the mobile phone. It was initially conceptualized as an irrational fear of spending time without a mobile phone, losing it, or being unable to use it [1], although more recent approaches describe it less as a specific phobia in the strict clinical sense and more as a behavioral syndrome linked to technological dependence, emotional distress, and anxiety when interaction with the device is interrupted [2, 3, 4]. In adolescent populations, nomophobia is commonly organized around dimensions such as anxiety about being unable to communicate, loss of connectivity, inability to access information immediately, and discomfort when the convenience provided by the device is no longer available [5]. These dimensions may be expressed through nervousness, irritability, obsessive thoughts, physiological activation, or compulsive checking behaviors [6]. From this perspective, the mobile phone does not merely operate as an instrumental device, but as a resource that supports communication, social presence, emotional reassurance, and access to immediate feedback.

However, nomophobia should be distinguished from other related but non-equivalent constructs, particularly digital control and emotional dependence. Digital control refers to interpersonal behaviors aimed at monitoring, supervising, or demanding access to another person’s online activity. Continuous smartphone use makes it possible to observe peers’ social activity in real time, know their availability, check connection status, confirm message reads, and anticipate replies, shaping relational dynamics based on immediacy and surveillance. These practices, often normalized in adolescent relationships, have been associated with emotional dependence, relational anxiety, and interpersonal conflict [7, 8, 9, 10]. Nevertheless, digital control is not the same as nomophobia: whereas nomophobia refers primarily to distress associated with the absence, loss, or unavailability of the mobile phone, digital control refers to relational practices of monitoring or demanding access to another person’s digital presence [10]. Emotional dependence, in turn, refers to a broader relational pattern characterized by excessive need for approval, reassurance, proximity, or validation, and has been associated with interpersonal vulnerability, anxiety, and problematic mobile or Internet use [11, 12]. These constructs may overlap in adolescent digital interactions, but they should not be treated as interchangeable. Accordingly, the present study does not assume that nomophobia directly causes digital control, emotional dependence, or child-to-parent violence (CPV). Rather, it examines whether six Adolescent Social Media and Internet Addiction Risk Scale Reliability and Validity (ERA-RSI) items related to mobile-phone-related anxiety, reassurance, and dependence show a coherent pattern of association within a specific justice-involved sample.

This distinction is especially relevant given the extensive literature on digital control in adolescent dating relationships, where expectations of immediacy, availability, and digital reassurance have been linked to technology‑mediated conflict and coercive dynamics [13, 14, 15, 16]. Although the present study does not assess digital violence or controlling behaviors, this body of research provides a relational context in which mobile‑phone‑related anxiety may acquire particular psychological relevance during adolescence.

CPV constitutes a highly specific and clinically relevant context in which to examine mobile-phone-related distress. CPV is commonly defined as repeated psychological, physical, or economic aggression exercised by children toward their parents with the aim of obtaining power, control, or domination [17, 18]. Previous research has shown that conflicts associated with the imposition, withdrawal, or limitation of parental rules may act as triggers for aggressive episodes toward parental figures, particularly among adolescents with difficulties in emotion regulation, low frustration tolerance, or a high need for control [19]. In this context, the mobile phone may acquire particular salience within family dynamics, as it concentrates communication, autonomy, peer affiliation, and perceived privacy.

For this reason, the present study adopts a limited item-level exploratory approach. Instead of attempting to validate the complete structure of nomophobia or to establish causal links between nomophobia, emotional dependence, digital control, and CPV, it examines the internal pattern of associations among six ERA-RSI items [20] related to nomophobia in adolescents serving court-ordered measures for CPV. This focus is useful because global scale scores may obscure differences between item contents: some items may reflect anxiety about immediate replies or lack of interaction, whereas others may refer to perceived safety, privacy, or instrumental uses of the device. Examining these item-level associations may therefore help identify which contents are more closely connected within this specific sample. However, this approach remains preliminary and correlational. The study does not directly measure emotional regulation, interpersonal monitoring, digital violence, or family conflict episodes, and its cross-sectional design does not allow causal or directional interpretations. Accordingly, the aim is not to describe the full structure of nomophobia, but to provide a cautious exploratory analysis of how selected nomophobia-related indicators are associated in a specific group of justice-involved adolescents.

## Methods

### Participants and Procedure

A cross-sectional study was conducted with 53 adolescents, 62.3% boys and 37.7% girls, aged between 14 and 19 years (M = 16.96; standard deviation (SD) = 1.20), who were serving court-ordered measures for CPV at the El Laurel Juvenile Justice Execution Centre in Madrid, the only specialized custodial facility for this type of violence in the region. According to Article 14.1 of Organic Law 5/2000 on the Criminal Responsibility of Minors, adolescents who reach legal adulthood while serving a judicial measure may remain within the juvenile justice system until the objectives of the sentence have been fulfilled, which justifies the inclusion of 19-year-old participants.

It is important to highlight that the target population of the present study was not the general adolescent population, nor the overall population of adolescents involved in juvenile justice, but the finite and highly specific population of adolescents placed in custodial measures for CPV-related offending in the Community of Madrid. This distinction is methodologically important. During 2024, the Community of Madrid implemented 543 custodial juvenile justice measures. In the same period, 64 minors were placed in custodial measures due to upward family abuse, that is, CPV, according to the Annual Report of the Agency for the Re-education and Reintegration of Juvenile Offenders [21]. Therefore, the present sample of 53 participants represents 82.8% of the annual target population. Rather than constituting a conventional small convenience sample, the study approaches a near-population design within a restricted forensic-psychosocial population.

To assess the adequacy of the sample size, a finite population correction was applied because the target population was clearly bounded and known in advance. The study did not aim to estimate parameters for the general adolescent population, but for a specific finite population. This approach is particularly suitable when the sample represents a high proportion of the accessible population, as in the present study, where 53 of the 64 eligible adolescents were included. Therefore, the finite population correction provides a proportionate assessment of sample size adequacy for this exploratory study. Assuming the most conservative scenario for proportions, *p* = 0.50 and *q* = 0.50, and a finite population of *N* = 64, the required sample size was estimated according to the following formula:

(1) $$n=\frac{N\,Z^{2}\,p\,q}{e^{2}\,\left(N\,–\,1\right)+Z^{2}\,p\,q}$$

Where *N* is the finite population size, *Z* is the critical value associated with the selected confidence level, *p* and *q* are the expected population proportions, and *e* is the admissible margin of error. It is relevant to consider what the literature emphasizes in connection with the sample size determination that should not be reduced to a single fixed rule, but should jointly consider population size, confidence level, margin of error, expected effect size, study design, feasibility, and accessibility of the target population [22, 23]. This is especially relevant in studies involving institutionally bounded or hard-to-reach populations, where the construction of conventional probability samples is often difficult and where findings should be interpreted within the limits of the target population rather than generalized to broader populations [24].

Using *N* = 64, *p* = 0.50, *q* = 0.50, and a ±5% margin of error, the required sample sizes were 59 participants at the 99% confidence level, 55 participants at the 95% confidence level, and 52 participants at the 90% confidence level. The obtained sample of 53 participants therefore exceeds the minimum requirement for a 90% confidence level and falls very close to the 95% threshold. When the achieved margin of error is calculated for the actual sample size, the estimates are ±7.39% at 99% confidence, ±5.62% at 95% confidence, and ±4.72% at 90% confidence. These results indicate that the sample provides high coverage of the accessible annual target population within this specific juvenile justice setting. However, high coverage of a finite institutional population should not be interpreted as equivalent to high statistical power, correlation stability, or external representativeness. Given the small absolute sample size, the item-level correlations reported below should be interpreted as preliminary estimates that require replication in larger and multi-site samples. The use of a 90% confidence level was considered appropriate given the exploratory nature of the study, the finite size of the accessible population, and the difficulty of recruiting adolescents subject to custodial measures for CPV. In exploratory studies, the primary aim is not to obtain definitive population estimates for broad generalization, but to identify theoretically meaningful patterns, generate hypotheses, and provide preliminary evidence in under-researched populations. Accordingly, the 90% confidence level should not be interpreted as a lower methodological standard, but as a proportionate decision aligned with the exploratory purpose of the study, the high coverage of the target population, and the restricted accessibility of the population under investigation. Recent methodological work supports the view that sample size decisions must be adapted to the research design, inferential purpose, and feasibility conditions of the study, particularly when the population is finite or difficult to access [22, 23, 24].

The Annual Report of the Spanish Public Prosecutor’s Office has also warned of a worrying intensification of violent behaviors carried out by minors, a phenomenon reported almost unanimously by juvenile prosecution sections in several Spanish territories, including Tenerife, Madrid, Alicante, Seville, the Balearic Islands, Ourense, Valladolid, and Barcelona [25]. In particular, this report highlights an increase in offenses linked to intrafamily violence and to the use of social networks as a means of aggression or control, as well as growing concern regarding the criminological incidence of offenses committed by minors under 14 years of age, many of which occur within the family context. These data reinforce the relevance of examining mobile phone-related anxiety, digital dependence, and interpersonal control dynamics in adolescents involved in CPV-related offending.

Data collection took place in two periods, from April to June 2023 and from February to April 2024, using convenience sampling within the accessible target population. The centre’s psychologists at El Laurel administered the scale in paper format on site, and responses were subsequently coded for quantitative analysis. Participation was voluntary and anonymous. The study was approved by the Ethics Committee of the Faculty of Education of the Universidad a Distancia de Madrid (N03/22_23) on 27 February 2023 and by the Agency of the Community of Madrid for the Re-education and Reintegration of Juvenile Offenders on 16 February 2023. The regulatory basis for conducting the study derives from the formal authorisation granted by this regional authority, which oversees juvenile justice centres, thereby permitting data collection within the El Laurel Juvenile Justice Execution Centre. The collaborative recruitment of participants was carried out in coordination with the centre’s professional team, in accordance with institutional protocols and under the supervision of the responsible authority. The study was conducted in accordance with the Declaration of Helsinki.

Before data collection, the technical team of the centre, composed of psychologists and social workers, identified the adolescents who were potentially eligible for participation during the two data-collection periods. Inclusion criteria were: (a) being subject to a court-ordered custodial measure for CPV; (b) being present at the centre during the data-collection period; (c) having sufficient Spanish-language competence to understand the questionnaire and the consent or assent procedure; and (d) providing written assent, together with written informed consent from parents or legal guardians when the participant was under 18 years of age. Adolescents who had reached legal adulthood while serving a judicial measure were also eligible and signed their own informed consent forms. Exclusion criteria were: (a) insufficient language competence to complete the questionnaire reliably; (b) legal or judicial circumstances preventing participation; (c) refusal to participate; and (d) missing responses in the six ERA-RSI nomophobia-related items selected for the present analysis.

### Instruments

The Risk Scale for ERA-RSI by Peris *et al*. (2018) [20] was used. This instrument assesses four dimensions through 29 items with a 4-point Likert response format: (1) addiction symptoms, (2) social use, (3) geek traits, and (4) nomophobia. In this sample, the scale showed adequate psychometric properties (Kaiser–Meyer–Olkin (KMO) = 0.90; χ^2^ = 6138.89; *p* < 0.001), explaining 46.5% of the total variance, with factor loadings ranging from 0.48 to 0.79. It is relevant to remark that the ERA-RSI is a broader instrument designed to assess risk of addiction to social networks and the Internet among adolescents, rather than a nomophobia-specific scale. The decision to use the ERA-RSI items was based on three reasons: first, the instrument had already been administered within the broader assessment protocol of the juvenile justice centre; second, the six items capture contents theoretically related to mobile-phone-related anxiety, reassurance, and perceived safety; and third, the aim of the study was exploratory and item-level, not diagnostic. Consequently, the findings are interpreted as associations among nomophobia-related indicators (as described in the next paragraph cocerning the validity of the scale) rather than as evidence about the full syndrome of nomophobia.

#### Validity of the ERA-RSI Scale

The KMO index and Bartlett’s test of sphericity yielded KMO = 0.90 and χ^2^(406) = 6138.89; *p* < 0.001, supporting the suitability of factor analysis. Because ERA-RSI items were answered using a 4-point Likert format, a polychoric correlation matrix was used, together with principal axis factoring (PAF) as the extraction method.

To determine the optimal number of factors, parallel analysis and the Minimum Average Partial (MAP) test were applied. Both methods converged on retaining four factors, consistent with the theoretical structure of the scale. An oblique Promax rotation was used because the dimensions are correlated. The factorial solution explained 46.5% of the total variance. Standardized factor loadings ranged from 0.48 to 0.79, and communalities (h^2^) ranged from 0.35 to 0.68. The resulting structure was coherent with the original theoretical model, with four clearly differentiated factors:

Addiction symptoms (9 items): emotional and behavioral manifestations derived from excessive use.

Social use (8 items): social bonding and dependence through networks.

Geek traits (6 items): a pattern of immersion in technological and playful activities.

Nomophobia (6 items): fear or anxiety when unable to access the device or connectivity.

Inter-factor correlations ranged from 0.42 to 0.63, supporting their relatedness while also indicating conceptual differentiation. No item showed cross-loadings above 0.30 on more than one factor, so the original 29 items were retained.

Analyses were conducted using IBM Statistical Package for the Social Sciences (SPSS), version 28.0 (Armonk, NY, USA). For the present study, we focused specifically on the six items of the nomophobia subscale:

Item 24: Safety/companionship through constant communication.

Item 25: Perceived safety when sending photos.

Item 26: Anxiety about immediate replies.

Item 27: Anger when separated from the device.

Item 28: Need for read-receipt confirmation.

Item 29: Restlessness when there is no interaction.

#### Data Analysis

Before calculating the item-level correlations, the distributional behavior of the six ERA-RSI nomophobia-related items was examined as a preliminary adequacy check for the planned exploratory analysis. This screening was necessary because Pearson correlations may be affected by severe departures from normality, restricted variability, extreme response concentration, and missing data, particularly when variables are measured using a four-point Likert response format. Accordingly, the inspection focused on determining whether the items showed sufficient variability and whether any item presented a clearly problematic distributional pattern, including severe non-normality, marked concentration of responses at one end of the scale, or missing responses in the six target items.

This assessment was treated as a pass/fail screening procedure for the exploratory correlational analysis rather than as a full descriptive objective of the study. The screening indicated that the six items met the minimum distributional conditions required for the planned analysis: no missing responses were identified in the target items, and none of the variables showed a distributional pattern severe enough to preclude the use of Pearson correlations as descriptive indices of observed item-level covariation. On this basis, Pearson correlation analysis was considered appropriate for the specific purpose of the present study, namely to provide a preliminary and easily interpretable examination of the bivariate association pattern among the six ERA-RSI nomophobia-related indicators.

Pearson correlations were therefore calculated among the six nomophobia-related items. All analyses were performed with SPSS v28.0, with statistical significance set at *p* < 0.05. Pearson coefficients were retained because the purpose of the analysis was descriptive and exploratory, not to estimate latent associations among underlying continuous variables. This decision should not be understood as assuming that the four-point Likert items are fully continuous or normally distributed variables, but rather as a proportionate analytic choice for an exploratory item-level study with a small, highly specific forensic sample. Consequently, the coefficients were interpreted cautiously and should be replicated in future studies using polychoric correlations and other ordinal-data methods.

Floor and ceiling effects were also examined as part of the distributional screening. For each of the six ERA-RSI nomophobia-related items, the number and percentage of participants selecting the lowest and highest response categories were calculated. A floor effect was defined as 70% or more of responses concentrated in the lowest response category, whereas a ceiling effect was defined as 70% or more of responses concentrated in the highest response category. This threshold was used as a descriptive criterion to identify marked response concentration at either end of the four-point scale. Proportions below this threshold were interpreted as indicating the absence of a clear floor or ceiling effect. These indicators were not used as exclusion criteria for the items, because the aim of the study was not to refine or validate a psychometric scale, but to describe the empirical behavior of a predefined set of ERA-RSI nomophobia-related indicators.

## Results

Table 1 shows the correlation matrix among the ERA-RSI nomophobia items. A pattern of significant intercorrelations can be observed, revealing two clear symptom groupings:

**Table 1.** **Pearson correlation matrix among items ERA-RSI_24–ERA-RSI_29**.

Item	24	25	26	27	28	29
ERA-RSI_24	1.00	**0.47**	**0.28**	**0.40**	**0.43**	**0.29**
		(*p* < 0.001)	(*p* = 0.04)	(*p* = 0.003)	(*p* = 0.001)	(*p* = 0.03)
ERA-RSI_25		1.00	0.23	**0.35**	**0.37**	0.16
			(*p* = 0.10)	(*p* = 0.01)	(*p* = 0.007)	(*p* = 0.24)
ERA-RSI_26			1.00	**0.55**	**0.54**	**0.69**
				(*p* < 0.001)	(*p* < 0.001)	(*p* < 0.001)
ERA-RSI_27				1.00	**0.64**	**0.69**
					(*p* < 0.001)	(*p* < 0.001)
ERA-RSI_28					1.00	**0.65**
						(*p* < 0.001)
ERA-RSI_29						1.00

Note: Significant correlations are shown in bold (two-tailed *p*-values are reported). The main diagonal (1.00) is omitted as trivial, and the upper half of the matrix is symmetric to the part displayed. ERA-RSI, Adolescent Social Media and Internet Addiction Risk Scale Reliability and Validity.

Items 26, 28, and 29 showed particularly strong positive correlations, ranging from r = 0.54 to r = 0.69. These items refer to anxiety about immediate replies, the need for read-receipt confirmation, and restlessness when there is no interaction. This pattern suggests that, in this sample, indicators related to immediate availability and interactional confirmation tended to covary more closely with one another than with some of the other nomophobia-related items.

Item 27, referring to anger when separated from the mobile phone, was also strongly associated with the items related to immediate replies, confirmation, and lack of interaction. Its correlations with these items ranged from r = 0.55 to r = 0.69, and its association with the need for read-receipt confirmation was r = 0.64. These results indicate that, in this sample, separation-related anger was empirically linked to indicators of availability and interactional reassurance. However, this association should not be interpreted as evidence that digital social anxiety translates into aggression, since the study did not assess causal direction, emotional regulation, or behavioral episodes of aggression related to mobile phone restriction.

Items 24 and 25 showed weaker associations with the remaining items. Their correlations with the other items ranged from r = 0.28 to r = 0.47, suggesting that contents related to perceived safety, companionship, or privacy through mobile phone use were less strongly associated with the items focused on immediate replies, read-receipt confirmation, lack of interaction, and separation from the device. Item 25, “I think it is safer to send a photograph by mobile phone than to post it on other social networks”, showed the weakest correlational pattern, with only two significant correlations, namely with Items 24 and 27. This may indicate that perceived digital privacy or safety represents a more specific item content within the six-item set, although this interpretation remains descriptive and should not be understood as evidence of a separate dimension.

Overall, the correlation matrix among items ERA-RSI_24–ERA-RSI_29 showed a pattern in which Items 26, 27, 28, and 29 presented higher and more consistent associations, with correlations approximately ranging from r = 0.54 to r = 0.69, whereas Items 24 and 25 were moderately related to each other, r = 0.47, and showed weaker associations with the rest of the items. This pattern may suggest differences in the empirical proximity of item contents, but it does not allow conclusions about the latent dimensionality or internal structure of nomophobia.

In addition, a descriptive association index was computed as the sum of the absolute values of each item’s correlations with all other items. This index was used only to summarize the relative degree of bivariate association of each item within the observed correlation matrix. Results showed that Item 27, anger when separated from the device, and Item 28, need for read-receipt confirmation, had the highest values, C = 2.63 in both cases, followed by Item 29, C = 2.48, and Item 26, C = 2.29. In contrast, Item 24, C = 1.87, and especially Item 25, C = 1.58, showed lower values. These results indicate that Items 27 and 28 had the highest cumulative association with the remaining items in this sample, whereas Items 24 and 25 showed comparatively lower cumulative associations.

## Discussion

The results suggest that Items 26, 27, 28, and 29 were more strongly associated with one another than Items 24 and 25. These items refer to anxiety about immediate replies, anger when separated from the mobile phone, need for read-receipt confirmation, and restlessness when there is no interaction. In this sample, contents related to availability, interactional confirmation, and discomfort when access or interaction is interrupted tended to covary more closely. This interpretation is consistent with previous research linking nomophobia and problematic mobile phone use with social anxiety, emotional discomfort, and the use of digital interaction as a source of reassurance or self-regulation [26, 27]. However, these findings should not be interpreted as evidence of a latent factor, symptom network, central component, or stable structure of nomophobia. The analysis was based on bivariate item-level correlations and did not apply factor analysis, network analysis, or any other structural model.

The positive association between anger when separated from the device and items related to availability, confirmation, and lack of interaction may also be read in light of previous work on emotion dysregulation in justice-involved adolescents [28]. However, the present study did not directly assess emotion regulation, frustration tolerance, impulsivity, parental rule enforcement, digital control behaviors, or aggressive episodes related to mobile phone restriction. Therefore, it cannot be concluded that nomophobia causes emotional dysregulation, that mobile phone restriction triggers aggression, or that these item associations explain CPV. Explanations involving family conflict, parental control, or aggressive escalation should be treated as hypotheses for future research.

Items 24 and 25 showed weaker associations with the remaining items. These items refer to perceived safety, companionship, or privacy when using the mobile phone. This may indicate that they capture contents less directly related to immediate interactional discomfort than items focused on replies, confirmation, lack of interaction, and separation from the device. This interpretation is compatible with models that distinguish affectively driven problematic technology use from more instrumental forms of use [29]. However, weaker correlations should not be interpreted as evidence of a separate dimension or conceptual independence. They only indicate weaker bivariate associations within this six-item set.

These findings are also limited by the measurement scope of the study. The six items analyzed belong to the nomophobia dimension of the ERA-RSI, but the ERA-RSI is a broader instrument designed to assess risk of addiction to social networks and the Internet among adolescents. Therefore, the findings should not be equated with the complete structure of nomophobia or with results obtained from nomophobia-specific instruments. They only describe the observed association pattern among six ERA-RSI indicators related to mobile-phone-related anxiety, reassurance, perceived safety, and interactional discomfort. Future studies should test whether similar patterns appear when using validated nomophobia-specific instruments and methods designed for ordinal data.

Several limitations should be noted. First, the study used a cross-sectional and self-report design, which prevents conclusions about temporal order, causal direction, or developmental pathways . Second, although the sample represents a high proportion of the accessible annual target population in this juvenile justice setting, the absolute sample size remains limited for item-level correlations. Third, the sample came from a single specialized juvenile justice centre and did not include a comparison group. Therefore, it is not possible to determine whether the observed pattern is specific to adolescents involved in CPV, to justice-involved adolescents more broadly, or to adolescents with other psychosocial difficulties. Fourth, the analysis did not adjust for demographic, family, or clinical variables such as age, gender, socioeconomic status, family structure, previous psychological conditions, impulsivity, or aggression. Unmeasured heterogeneity may therefore have influenced the associations.

The findings should also be interpreted with caution regarding generalizability. The study focuses on adolescents serving court-ordered measures for CPV in a specialized juvenile justice centre. This is a difficult-to-access population that is underrepresented in research on nomophobia and problematic mobile phone use, which has mainly focused on school or community samples. At the same time, the specificity of the sample limits external validity. The findings should not be generalized to the general adolescent population or to all adolescents involved in family conflict. They may offer preliminary insight for comparable forensic-psychosocial contexts, but replication in larger, multi-site samples is needed.

Future research should replicate these findings with larger and multi-site samples, comparison groups, validated nomophobia-specific scales, longitudinal designs, and ordinal-data methods. It should also include direct measures of emotional regulation, impulsivity, family conflict, parental rule enforcement, digital control behaviors, and aggressive episodes associated with mobile phone restriction. Such designs would help determine whether the association pattern observed here is specific to CPV-involved adolescents, reflects broader psychosocial vulnerability, or represents a more general feature of adolescent mobile-phone-related distress.

## Conclusions

This exploratory study examined item-level associations among six ERA-RSI nomophobia-related indicators in adolescents serving court-ordered measures for CPV. The results showed positive associations among the six items. Items related to immediate replies, read-receipt confirmation, lack of interaction, and anger when separated from the mobile phone were more strongly associated with one another than items referring to perceived safety when communicating or sending photographs.

These findings should be interpreted as preliminary descriptive evidence from a specific justice-involved sample. The study does not assess the full structure of nomophobia, does not include a control group, and does not establish causal relations between mobile-phone-related anxiety, interpersonal control, emotional dysregulation, or CPV. Future research should replicate these findings with larger and multi-site samples, comparison groups, validated nomophobia-specific scales, longitudinal designs, control variables, and ordinal-data methods.

## Availability of Data and Materials

The datasets analyzed during the current study are available from the corresponding author on reasonable request.
